# Influence of root distribution patterns on soil dynamic characteristics

**DOI:** 10.1038/s41598-022-17828-2

**Published:** 2022-08-04

**Authors:** Shusen Liu, Jun Li, Xiaodong Ji, Yi Fang

**Affiliations:** 1 Department of Civil Engineering, Beijing Forestry University, Beijing, 100083 People’s Republic of China; 2 Key Laboratory of State Forestry Administration on Soil and Water Conservation, Beijing Forestry University, Beijing, 100083 People’s Republic of China; 3 National Institute of Natural Hazards, Ministry of Emergency Management of China, Beijing, 100083 People’s Republic of China

**Keywords:** Civil engineering, Engineering, Environmental sciences, Environmental impact, Natural hazards

## Abstract

Slopes along the highway and railway routes are subjected to not only static loads but also dynamic loads generated by vehicles and trains. The induced excessive deformation potentially poses a threat to slope stability. In terms of the extensive application of ecological slope protection, plants play a critical role in slope stability, as the roots can enhance the shear strength of the soil. This study aims to investigate the influence of different root distribution patterns on the dynamic characteristics induced by cyclic loading. By conducting a group of dynamic triaxial tests, the results indicate that the root system can significantly enhance the liquefaction resistance of the soil when the soil is subjected to lower dynamic loads, and the cross arrangement has a better-reinforced effect than the mixed arrangement. The reinforced effect was not obvious when the soil was subjected to a dynamic load with a larger stress amplitude. In addition, based on the validation of the seed model, a new pore water pressure development model was proposed according to the test results. Overall, the research provides a new model and some innovative observations to better understand the dynamic behavior of root-reinforced soil.

## Introduction

The continuous expansion of the road and railroad network in China has induced a large amount of artificial slope construction along the route. As slopes suffer from complex loads, such as gravity, rainfall, underground water, and traffic loads, the induced deformation may lead to slope instability, threatening the normal operation of people's lives and resulting in serious property damage and casualties^1,2^. Compared with traditional engineering slope protection, ecological slope protection technology, which has been widely used, has superior protection effects on stabilizing shallow soil, preventing shallow landslides, and beautifying the environment^3–7^.

Slope bioengineering has a significant protective effect on the ecological environment, and vegetation has a complex effect on the stability of slopes. Numerous studies have proposed that plant roots can strengthen the soil body mainly through the reinforcing effect of fine roots^8^ and the anchoring effect of thick roots^9^. In the shallow soil layer, the fine roots evenly penetrated the soil body and tightly entwined with the soil body to play a reinforcing role, enhancing the shear strength of the slope soil. By contrast, thicker and deeper roots penetrate the soil body as anchors to improve friction with the soil body, thus strengthening the soil body and enhancing its ability to resist damage^10–13^. For fine roots, taking root-reinforced soils as one kind of composite material can provide access to a breakthrough for the study of the reinforcement effect of roots on slope soil. The shear strength occupies a dominant position to reveal the root reinforcement mechanism. With the improvement of the shear strength test method, many meaningful results about the root reinforcement effect have been proposed. First, direct shear tests, as one kind of traditional strength test, have been widely used to investigate the reinforcement effect of different plant roots, and the results showed that the shear strength of root-reinforced soil has improved compared with that of plain soil by root enhancing the soil cohesion^14,15^. Data obtained by subsequent researchers through direct shear tests showed that root geometry^16–19^ (root diameter and root length) and distribution characteristics^20,21^ (root density and root distribution angle) had a significant effect on the shear strength of the soil. Second, triaxial compression tests are also commonly conducted to study the properties of root-reinforced soils. Compared with direct shear tests, triaxial compression tests can provide more complex loading modes and better reflect the true stress–strain relationship of the specimen^22,23^. Regarding the study of root distribution properties, Zhang et al.^22^ and Lian et al.^23^ investigated the effects of three analytical distributions, vertical, horizontal, and cross, on the shear strength of root-soil complexes by triaxial compression tests and suggested that the enhancement effect of cross root distribution was optimal. Wang et al.^24^ tested four types of herbaceous root systems: vertical, inclined, herringbone (crossed), and zigzag (crossed plus vertical) distribution and found that the zigzag root system method of distribution had the best enhancement of shear strength of the root-soil complex. In summary, plant roots have an obvious reinforcement effect on the soil, and the effect is significantly affected by the physical properties and spatial distribution of the root system. Nevertheless, most of the research is limited to static loading but ignores complex loads, such as dynamic loading, which can also induce deformation of the slope. It is essential to study the dynamic response of root reinforced soil, especially for the deformation behavior, to provide a theoretical foundation for slope deformation control.

The long-term cyclic load has the potential to cause deformation or damage to slope soil structures, resulting in slope instability^25–27^. Current research on the dynamic characteristics of soil under cyclic loading is mainly carried out with homogeneous mass soil. Tang et al.^28^ and Li et al.^29^ investigated the dynamic properties of powdered clay soils under cyclic triaxial tests, and some critical influence factors, such as the loading frequency and the dynamic stress amplitude, were taken into consideration. The results demonstrated that the dynamic elastic modulus of the soil decreased with increasing dynamic stress amplitude and increased with increasing loading frequency. Similarly, Luo and Miao^30^ conducted a prediction study on the dynamic creep strain and settlement of soft soil under the subway by simulating the subway vibration load. In addition, more influencing factors (number of loading cycles, initial static flexural stress, confining pressure, initial static shear stress, stress path, and soil density) were also considered for further research on the soil strain behavior and the accumulated pore water pressure^31,34,35^. Based on numerical simulation of traffic loads, some scholars have established the cumulative deformation model and hysteresis curve model of soil under cyclic loading by considering the dynamic stress amplitude, vibration frequency, dynamic partial stress, and other influencing factors^32,33^. Not just focusing on homogeneous soil, some scholars have studied geosynthetic-reinforced soil to verify that reinforced soil has a reinforcing effect on the soil under cyclic loading^36–38^. However, BS^39^ employed train-controlled cyclic simple shear tests to investigate the effects of fiber content, length, and relative density on the effectiveness of sand soil improvement and concluded that fiber inclusion increases the resistance of the soil to liquefaction. The study concept of composite materials applied to the reinforcement effect is also suitable for root reinforced soil. At present, research on soil under cyclic loading mainly focuses on homogeneous soil and geosynthetic-reinforced soil, while research on root-reinforced soil is still in the initial stage and lacks relevant research.

In this paper, a common species of slope protection plant (*Pennisetum alopecuroides (Linn.) Spreng*) in northern China was selected as the reinforcement material. Through dynamic triaxial tests, the influence of root distribution patterns on the dynamic characteristics of root-reinforced soil under cyclic loading was studied by controlling the dynamic stress amplitude and root distribution patterns. The stress–strain relationship, dynamic strength, and pore pressure change law of root-reinforced soil were also discussed in the paper. A new air pressure model is proposed.

## Material and test method

### Test material

**Figure 1:**
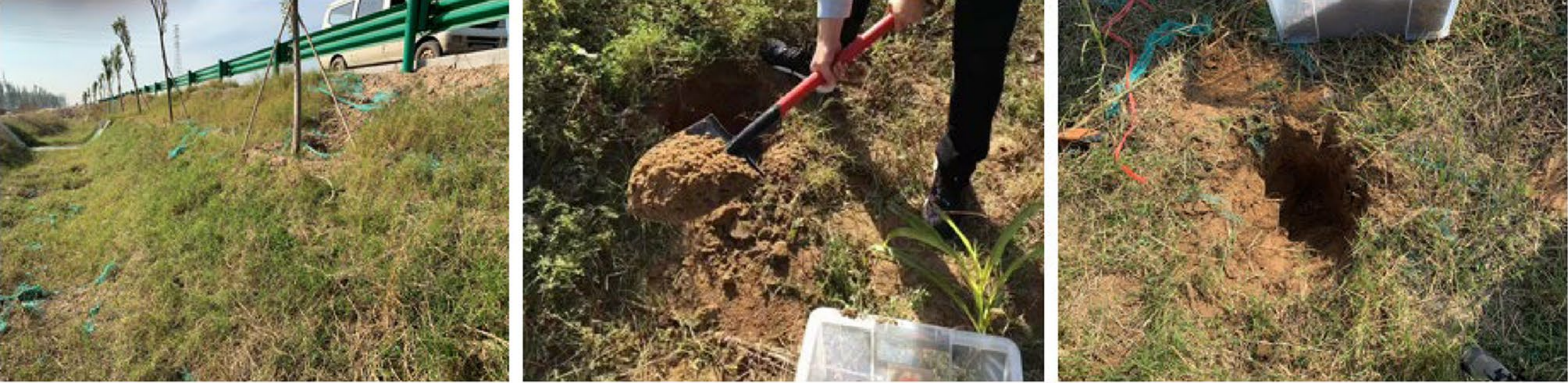
Raw soil extraction process.

The soil samples were obtained from the slope of national highway G101 in the Huairou district (40° 19′ 42′ N, 116° 41′ 45ʺ E) of Beijing. The regional climate is warm-temperate and semihumid. The average annual temperature ranges from 9 to 13 °C, and the average annual precipitation ranges from 600 to 700 mm, mainly concentrated in June to August. As the liquid limit of this soil is 31%, the plastic limit is 17%, and the plasticity index is 14, the type of this soil is identified to be silty clay according to the GB50007—2002 specification. Pictures of the original soil samples are shown in Fig. 1. Other physical and mechanical properties of the soil sample are illustrated in Table 1. Geotechnical parameter testing is strictly conducted in accordance with the Geotechnical Test Method Standard (GBT50123-1999).

**Table 1:** Physical and mechanical properties of the soil sample.

Indicator items	Density (g/m^3^)	Dry Density (g/m^3^)	Specific gravity of soil particle	Water content (%)	Optimum water content (%)	Maximum dry density	Porosity ratio (g/m^3^)	Saturation (%)
–	1.66	1.41	2.72	15.5	1.69	1.72	0.93	81

A representative common herb, fountain grass (*Pennisetum alopecuroides (Linn.) Spreng.*), was selected as the reinforced material for the experiments. The root collection method was performed following Boehm's "Root Parameters and Their Measurement"^40^. The plant roots were de-impurities treated, the morphological indexes were measured by the plant Analysis system (WinRHIZO), and the mean value was calculated as the morphological data of the root system^19,41^. The total root content of the plant was 8.79 g in the 0–50 cm soil layer. More specifically, in the 0–10 cm, 10–20 cm, 20–30 cm, 30–40 cm, and 40–50 cm soil layers, the corresponding root contents were 1.57 g, 1.72 g, 1.94 g, 1.81 g, and 1.75 g, respectively. The average root diameter was 0.7 mm.

### Specimen preparation

For the purpose of controlling the root distribution patterns, the samples for the tests are remolded soil. The sample preparation process was as follows: First, the soil taken from the site was dried in an incubator at a temperature of 105–110 °C for 8 h. Second, the dried soil sample was sieved and mixed with water. Finally, the prepared soil sample was put into a sealed bag and allowed to stand for 24 h. To acquire better vacuum saturation of the sample, according to the calculation results of the saturation formula and the conventional physical and mechanical properties of the actual soil, the dry density of the sample soil is set to $P_{d} = 1.42\;{\text{g/cm}}^{3}$, and the moisture content is set to $\omega = 18\%$. The mass of the soil sample required for each specimen was determined by the dry density and moisture content. According to the formula $m = P_{d} \left( {1 + \omega } \right)v$, the soil mass required for each sample was calculated. Based on the root scanning data, the average diameter was approximately 0.7 mm. To ensure uniform consistency across all specimens, a selected root of approximately 0.7 mm in diameter and 10 mm in length was added to each specimen.

**Figure 2:**
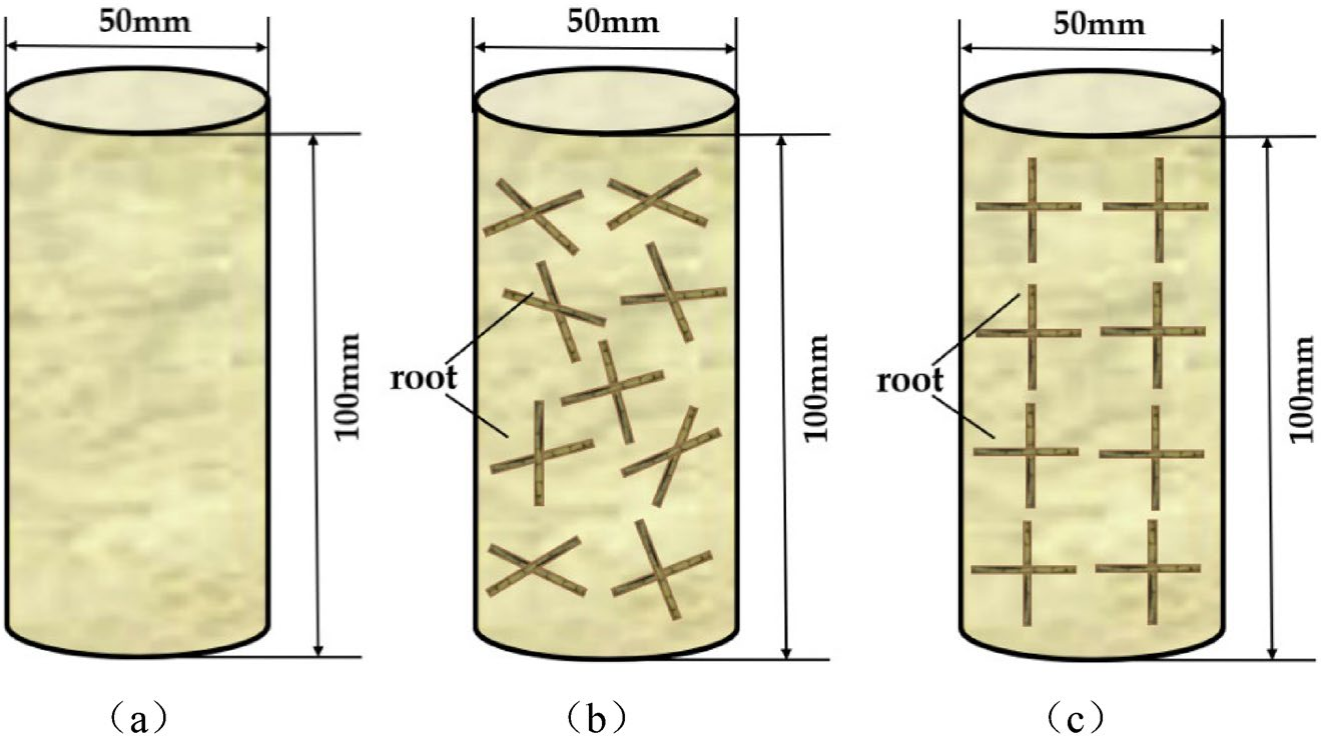
Three root distribution patterns. (**a**) Plain soil, (**b**) mixed reinforced soil, and (**c**) crossed reinforced soil.

Generally, the most frequently used root arrangement patterns for the current research are vertical, horizontal, crossed, and mixed arrangements. There is a consensus that crossed and mixed arrangement patterns have a better reinforcement effect than vertical and horizontal distribution patterns^20,21^. On this basis, we chose crossed arrangement and mixed arrangement, which are more closely matched to the actual distribution, for the study comparison. As shown in Fig. 2, three specimen types, plain soil, mixed reinforced soil, and crossed reinforced soil, were designed for the experiments to investigate the effect of root distribution on the deformation characteristics under cyclic loading. More specifically, for the mixed arrangement, without considering the distribution angle of the root system in the soil, all the roots added to the samples were mixed evenly and then divided equally into 5 layers. For each layer, the thickness of the compacted soil was approximately 20 mm as the total sample height was 100 mm. By contrast, for the crossed arrangement specimens, the root distribution angle was strictly limited to 0° and 90°. That is, as the total sample was divided into 5 layers, in each layer, the roots were kept in the vertical and horizontal directions with the same root amount. After that, the roots intersected at right angles, which are considered to be crossed distribution patterns.

### Test apparatus and test scheme

**Figure 3:**
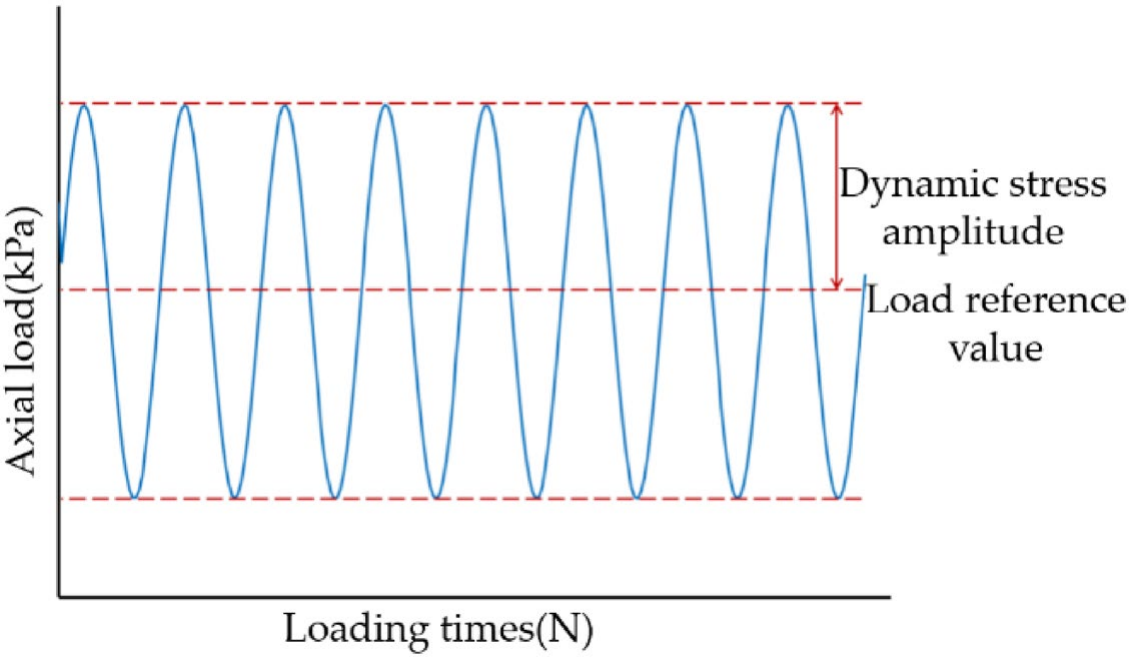
Single-stage cyclic loading diagram-typical sinusoidal cyclic loading.

As the research is based on the root reinforcement of slopes along the expressway, the impact of traffic loading needs more attention. Because the traffic load is a cyclic load with a long period, low frequency, and large influence range, which has been generally accepted^42^, our study uses a sinusoidal wave cyclic load to simulate the traffic loads, as shown in Fig. 3^43^. The cyclic triaxial tests were carried out on the HCA-100 dynamic hollow cylinder-TSH testing system (HCA-100) manufactured by GCTS Instruments (GCTS, USA). The height of the cylindrical specimens is 100 mm, while the diameter is 50 mm. Three main influencing factors, confining pressure, loading frequency, and dynamic stress amplitude, were considered to explore the dynamic behavior of the root-reinforced soil under cyclic loading. Based on the field monitor, the corresponding influence depth of the roadbed is 1.28–2.45 m^43–45^. Therefore, the confining pressure was set to 50 kPa. In terms of the railway and highway network, the traffic load frequency range is approximately 0.4–2.6 Hz, and thus the load frequency was set to 1 Hz. To study the influence of the dynamic stress amplitude, the stress values were set to 15 kPa, 20 kPa, and 25 kPa^46^. The tests stopped when the axial strain was larger than 15% or the sample reached the complete liquefaction state. There are 9 groups of consolidated-undrained dynamic triaxial tests, as shown in Table 2.

**Table 2:** Dynamic triaxial test scheme.

Soil sample	Dynamic stress amplitude (kPa)	Root content (g)	Number of vibrations
Plain soil	15	0	197
	20	0	84
	25	0	19
Mixed reinforced soil	15	1.5	816
	20	1.5	131
	25	1.5	25
Crossed reinforced soil	15	1.5	1569
	20	1.5	215
	25	1.5	36

Considering the complex loading applied on the slope soil, the dynamic loading should not be neglected, which may induce excessive deformation. More comprehensive research on the root reinforcement effect needs to consider different kinds of loading types, as conventional root-reinforced soil tests mainly pay attention to static loads. The purpose of this research is to reveal the dynamic properties of root-reinforced soils under dynamic loading. Through the test data obtained from the above test scheme, the dynamic strength, hysteresis curve, damping ratio, and pore water pressure variation law of the root-reinforced soil when liquefaction occurs under dynamic loading are derived. The results can provide a theoretical foundation for slope deformation control and slope stability considering the influence of dynamic loading.

## Data analysis methods

### Definition of soil liquefaction

Applying the dynamic load, the increase in pore water pressure and axial strain is accompanied by a reduction in the soil strength, and there is a corresponding residual strength related to different dynamic stresses. The point where the pore water pressure equals the lateral consolidation pressure and the corresponding dynamic strain expands significantly marks a visible loss of the residual strength of the soil. When the deformation is fully developed, the dynamic stress acting on the soil has only a very small fluctuation change, indicating that the soil is no longer able to withstand the action of dynamic stress, which is usually called the liquefaction phenomenon. The pore water pressure rises when liquefaction occurs in the soil as a result of the combined effect of the vibration generating pore pressure and drainage dissipating pore water pressure.

### Definition of dynamic strength

To date, there is no unique standard to define the dynamic strength of soils, which is variable under different test conditions. The current definition of dynamic strength can be divided into the following three categories: the first is the liquefaction criterion, i.e., the strength is determined when the pore water pressure is equal to the confining pressure; the second is the strain criterion, i.e., the strength related to the axial strain reaches 2.5%, 5%, and 10%; and the third is the limiting equilibrium criterion, i.e., the strength when the strain rate reaches its maximum value^47,48^. This test studies the effect of root reinforcement on the dynamic characteristics of soil, and the specimens all presented a liquefied state in the test, so the first type of liquefaction criterion is chosen. The dynamic strength curve demonstrates the relationship between the corresponding damage vibration number $N$ and the applied dynamic stress $\sigma_{d}$. When the dynamic pore pressure $u_{f}$ is equal to the consolidation lateral pressure, the state is considered to be a liquefaction state. Considering different dynamic stress magnitudes, the relationship between the damage vibration number *N* and stress amplitude $\sigma_{d}$ is commonly called the dynamic strength curve for liquefaction resistance.

### Damping ratio calculation method

**Figure 4:**
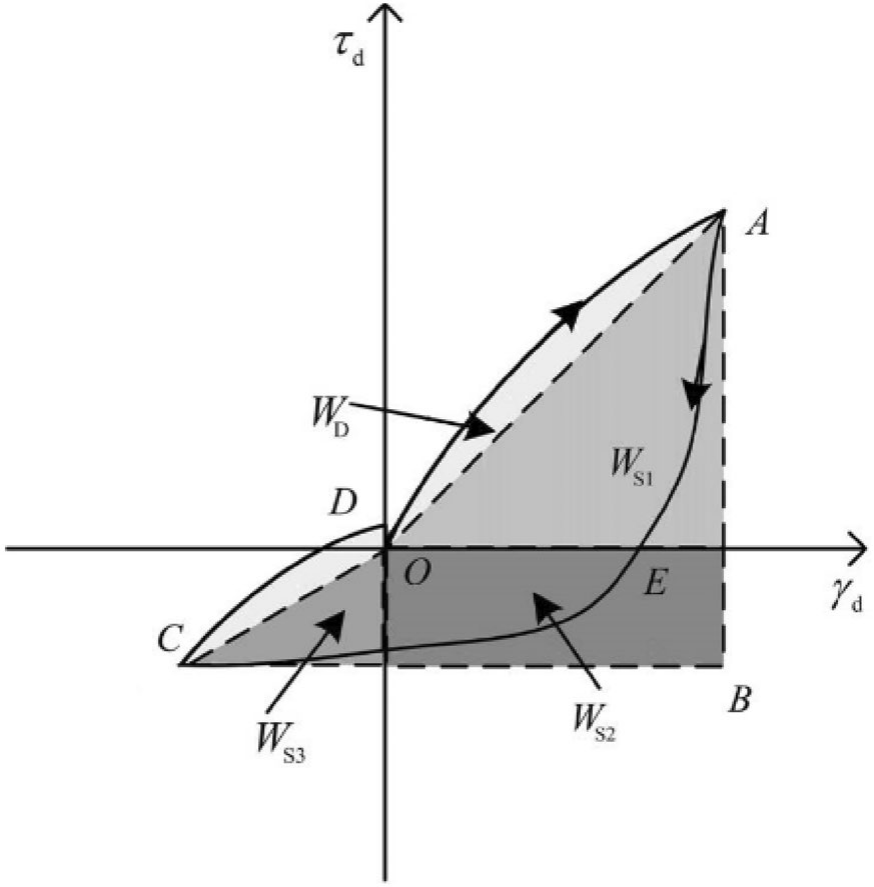
Diagram of the Kumar method principle^50^.

The damping ratio is a significant parameter for demonstrating the dynamic properties of soil, which indicates the decay form of soil vibration after excitation by describing the energy dissipation of soil during vibration. Derived from the test results, the damping ratios of different samples were calculated. This paper selects the Kumar method^49^ for the damping ratio calculation, as shown in Fig. 4 with formula 1. In formula 1, $W_{S1}$, $W_{S2}$, and $W_{S3}$ are the components of elastic strain energy stored in one cycle, and $W_{S} = W_{S1} + W_{S2} + W_{S3}$ and $W_{D}$ are the strain energy consumed in a cycle. Point O to point A is the positive loading stage, point A to point E is the positive unloading stage, point E to point C is the reverse loading stage, and point C to point D is the reverse unloading stage.

1 $$ \lambda = \frac{1}{4\pi } \times \frac{{W_{D} }}{{W_{S} }} = \frac{1}{4\pi } \times \frac{{W_{D} }}{{0.25\left( {W_{S1} + W_{S2} + W_{S3} } \right)}} $$

### Pore water pressure model

The development of pore water pressure under dynamic loading is an important aspect closely related to the change in deformation characteristics, and the study of the occurrence, development, and dissipation of dynamic pore water pressure is meaningful to better understand the liquefaction mechanism. Seed^53^ presented the following model (2) based on the dynamic triaxial test of saturated sandy soil with isotropic consolidation and without drainage. The formula as follows relates to the dynamic pore water pressure and the applied dynamic stress.

2 $$ \frac{u}{{\sigma_{0} }} = \frac{2}{\pi }{\text{arcsin}}\left( {\frac{N}{{N_{L} }}} \right)^{\frac{1}{2a}} $$

where $u$ is the pore water pressure, $\sigma_{0}$ is the initial effective stress, $N$ is the number of vibrations, $N_{L}$ is the corresponding vibration number reaching the liquefaction state, $a$ is the test constant, depending on the soil type and test conditions, and in most cases, $a = 0.7$.

In this paper, based on the test results, the seed model is validated, and a new pore water pressure model is proposed with a validation criterion of $R^{2}$. The goodness-of-fit ${ }R^{2} { }$ was introduced as a criterion to evaluate the effectiveness of the fit of the formula. Goodness-of-fit tests are statistical tests aiming to determine whether a set of observed values matches those expected under the applicable model. A larger $R^{2}$ indicates a better fit of the data points and the model, so a larger $R^{2}$ means a better fit.

### Plant handling statement

We strictly adhere to the IUCN Policy Statement on Research Involving Endangered Species and the Convention on Trade in Endangered Species of Wild Fauna and Flora. The plant selected for this study is fountain grass (Pennisetum alopecuroides (Linn.) Spreng.), which is a common slope protection plant in China. It is not a protected species, and our collection and research are in full compliance with national laws and international regulations.

## Results

### Dynamic strength

**Figure 5:**
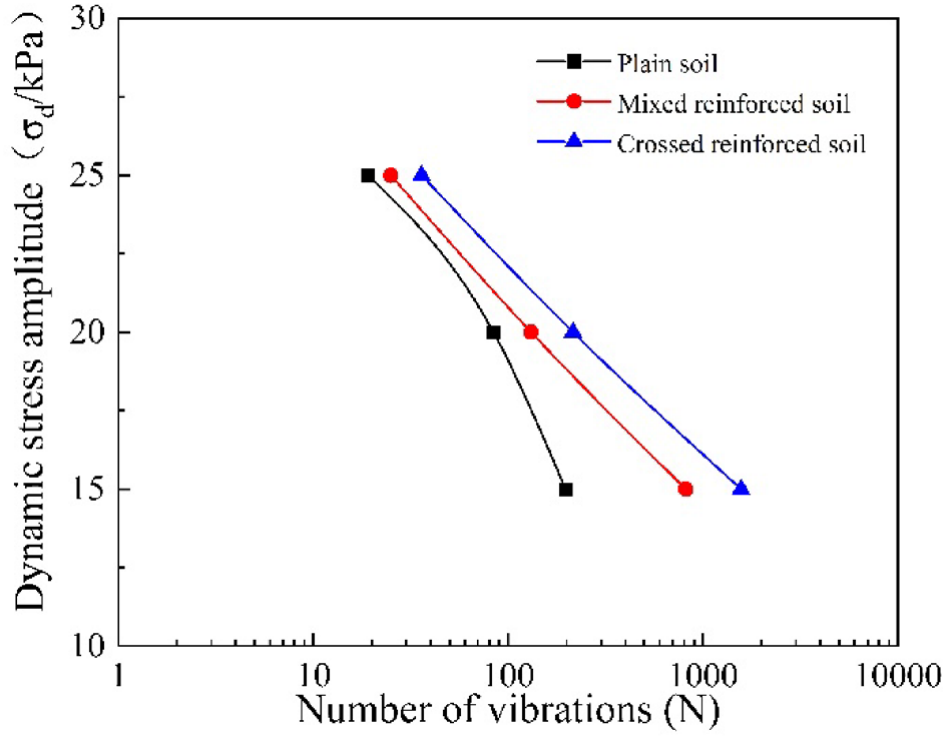
Dynamic strength curve-dynamic stress amplitude vs number of vibrations.

Figure 5 shows the dynamic strength curves of the three specimens under three dynamic stress amplitudes (15 kPa, 20 kPa, 25 kPa) with the same confining pressure (50 kPa) and loading frequency (1 Hz). There were a total of nine spots related to the nine groups of tests. From Fig. 5, the number of cyclic vibrations reaching liquefaction for plain soils with three dynamic loads is 197, 84, and 19. By contrast, the vibration number for the mixed arrangement soil is 816, 131, and 25, and the number is 1569, 215, and 36 for crossed arrangement soil. The number of cyclic vibrations required to reach the liquefaction state of reinforced soil is significantly larger than that of plain soil. Based on this, it is known that the root system has remarkably increased the soil dynamic strength, which represents the liquefaction resistance ability. The soil with the crossed distribution pattern possesses better dynamic strength than the soil with the mixed arrangement. Considering the dynamic stress amplitude, the root reinforced effects are diverse. More specifically, the effect of root reinforcement is quite obvious when subjected to a 15 kPa load, as the vibration number, which represents the liquefaction resistance for root mixed arrangement and cross arrangement soil, increased 314% and 696% compared to plain soil, respectively. When the dynamic stress amplitude reaches 20 kPa, the effect gradually decreases as the data points get closer. The vibration numbers of the mixed arrangement and crossed arrangement soils only grow by 56% and 156%, respectively. Until the amplitude of 25 kPa, the difference between normal soil and root reinforced soil is not significant, as the increased percentage is 31% and 89%, respectively. In summary, the root reinforcement effect is much better with a lower dynamic stress amplitude. In addition, for the two kinds of root reinforced soil, the number of vibrations in a logarithmic form has a clear linear relationship with the dynamic stress amplitude.

### Hysteresis curves

**Figure 6:**
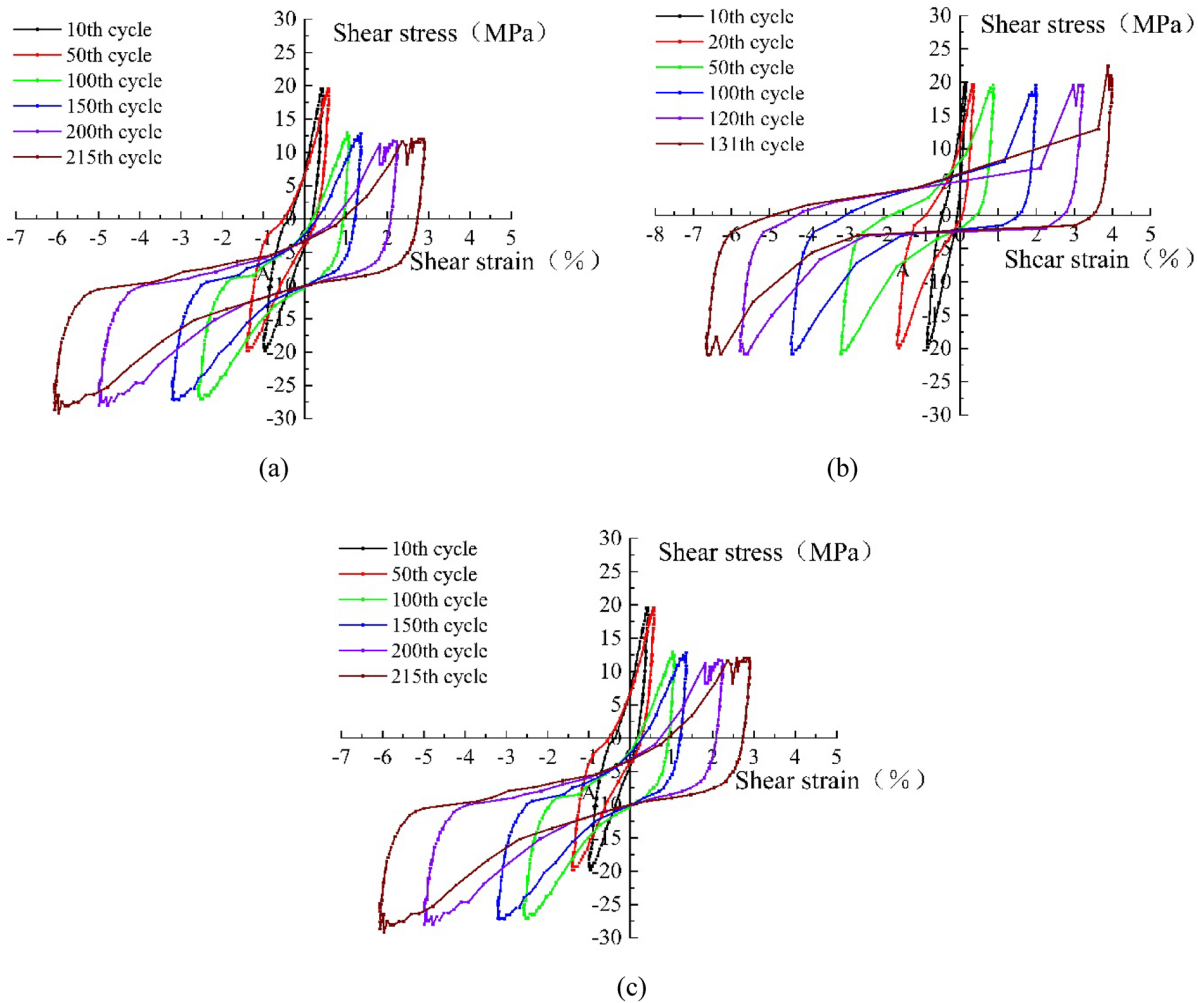
Hysteresis curves of the three different root distribution patterns of soils: (**a**) plain soil hysteresis curve, (**b**) crossed reinforced soil hysteresis curves, and (**c**) mixed reinforced soil hysteresis curves.

To investigate the influence of plant roots on the hysteresis behavior of soil, the hysteresis curves under 20 kPa dynamic stress amplitude are demonstrated in Fig. 6. Due to the remarkably different total number of cycle cycles in the three cases, it was not possible to select the same cycle interval. Thus, the cycle intervals were selected according to the specimen strains corresponding to 1%, 2%, 4%, 6%, 8%, and 10% axial strain. The areas of the hysteresis curves for the tenth cycle in Fig. 6a–c are 13.2, 10.6, and 9.2, respectively. When the axial strain reaches 2%, the area growth rates of the hysteresis curves are 176%, 182%, and 189%. When the axial strain reaches 4%, the growth rate of the hysteresis curve area is 107%, 102%, and 101%, respectively. The growth rate of the hysteresis curve area decreases at 6%, 8%, and 10%. As the deformation process gradually proceeds, the growth rate of the hysteresis curve area will show a trend of first increasing and then decreasing. Figure 6 shows that the position and shape of the hysteresis curve change considerably when the axial strain exceeds 2%. It is due to the structural rearrangement of the soil at this stage that the deformation of the specimen is not fully recovered, and with the gradual increase in the deformation, the specimen has irrecoverable plastic deformation.

In Fig. 6, when the number of vibrations increases, the positive shear strain value in the soil is smaller than the negative shear strain value, which is caused by the dilation strain being larger than the shear contraction strain. The reason for this is that the liquefaction effect of the soil grows continuously as the vibration load continues to increase into the soil, and the soil gradually changes from elastomeric to elastoplastic and finally to plastic in the process of this change. Therefore, it is impossible to return to the initial state at the beginning of liquefaction, which leads to shear expansion rather than shear contraction in the cycle.

**Figure 7:**
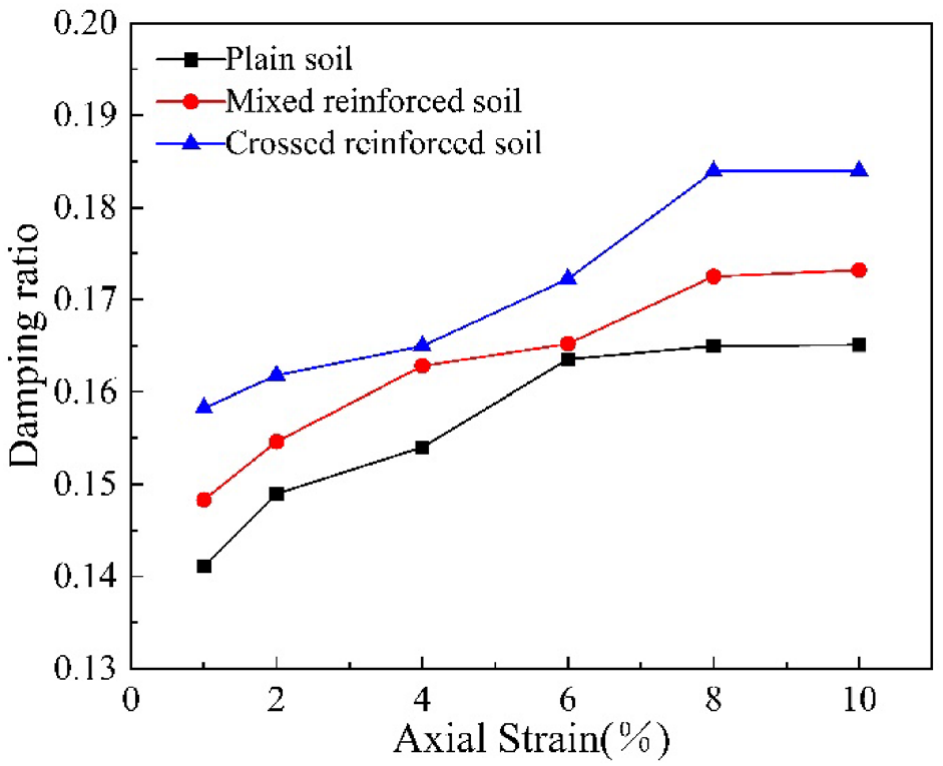
Curves of damping ratio veruss axial strain.

According to formula 1, the representative damping ratio vs dynamic strain relationship curves of the root-reinforced soil and the plain soil are shown in Fig. 7. The damping ratios of all three specimens increase at first and then tend to be stable at last. This is because, with the development of dynamic strain, the dissipation of energy inside the soil gradually accumulates, and the damping ratio of the soil increases. However, the dissipation of energy will become increasingly difficult with the development of dynamic strain, so the increase in damping ratio decreases and gradually tends to the maximum damping ratio. The damping ratio of the root-reinforced soil is significantly larger than that of the plain soil. The crossed reinforced soil has the largest damping ratio, followed by the mixed reinforced soil and plain soil. This indicates that suffering from the dynamic load, the plant roots in the soil can absorb energy, increase the damping ratio of the soil and enhance the ability of the soil to resist the cyclic load.

### Development pattern of dynamic pore pressure

**Figure 8:**
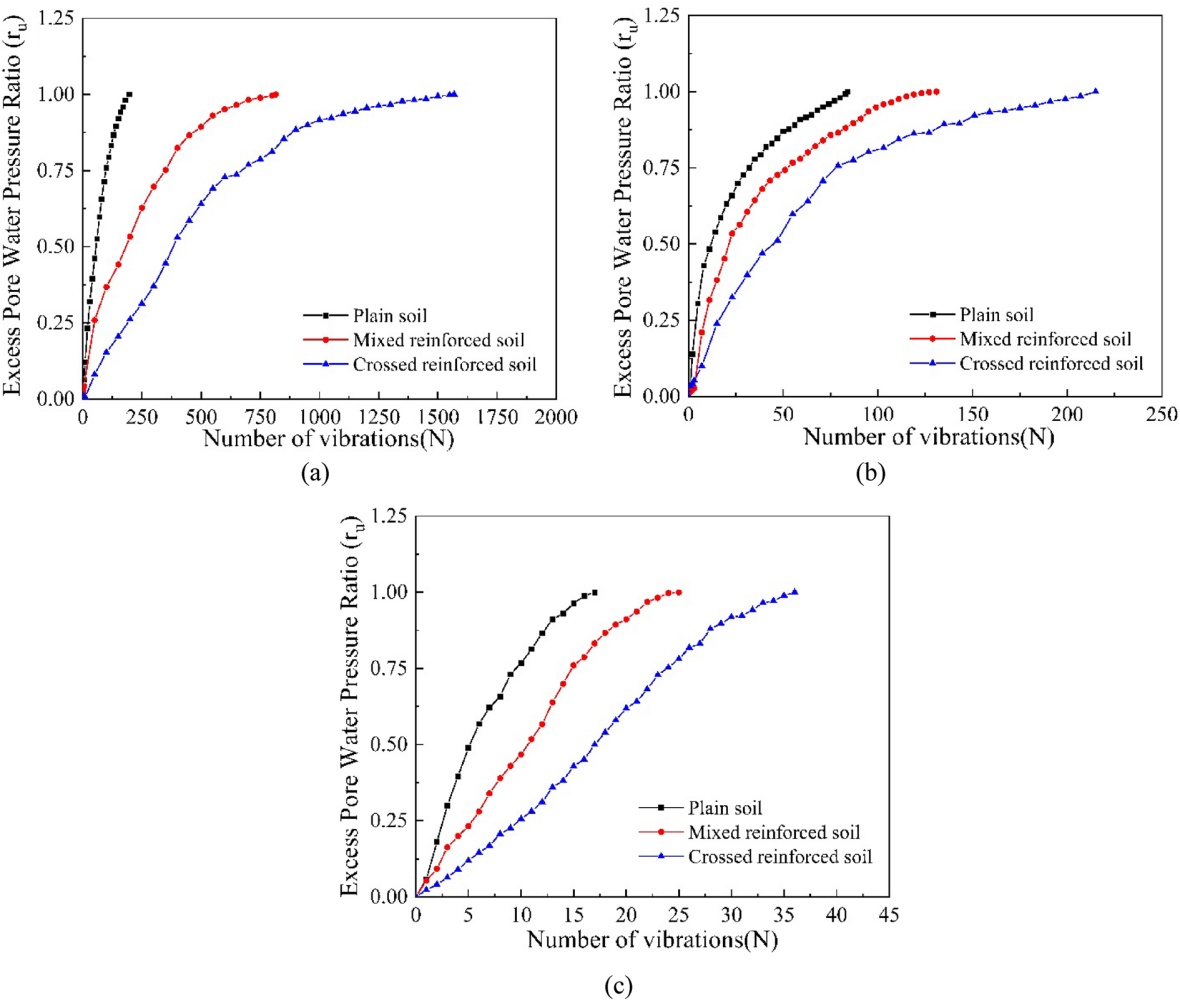
Pore water pressure ratio versus number of vibrations: (**a**) dynamic stress amplitude of 15 kPa, (**b**) dynamic stress amplitude of 20 kPa, and (**c**) dynamic stress amplitude of 25 kPa.

As mentioned above, the load frequency is set to 1 Hz, and the applied axial dynamic stress is 15 kPa, 20 kPa, and 25 kPa. According to the dynamic triaxial test data, the pore water pressure change process of the sample during liquefaction is sorted out, as shown in Fig. 8. The time-course curves of the pore water pressure ratio with the number of vibrations for samples with different root distribution patterns are shown in Fig. 8. The pore water pressure ratio ($r_{u}$) is defined as the ratio of excess pore water pressure to effective confining pressure. The soil loses strength and liquefies when $r_{u} = 1$. From Fig. 8, the pore pressure ratios of the soil samples under different dynamic stress conditions grow rapidly at first and then gradually tend to be stable. The pore pressure ratios of the plain soil increase most rapidly, followed by the mixed reinforced soil and the crossed reinforced soil. That is, under the same dynamic loading, plain soil is more prone to liquefication than root-reinforced soil. Specifically, the permeability of the soil in the test affects the effect of soil liquefaction; the smaller the permeability coefficient is, the less likely the soil is to undergo liquefaction^51,52^. Roots in the soil lead to greater cohesion in the soil^21–23^, reducing the permeability of the soil and making the soil less susceptible to liquefaction. This suggests that the root system can slow down the growth rate of soil pore water pressure and has better resistance to liquefaction by cross arrangement.

### Pore water pressure models

**Figure 9:**
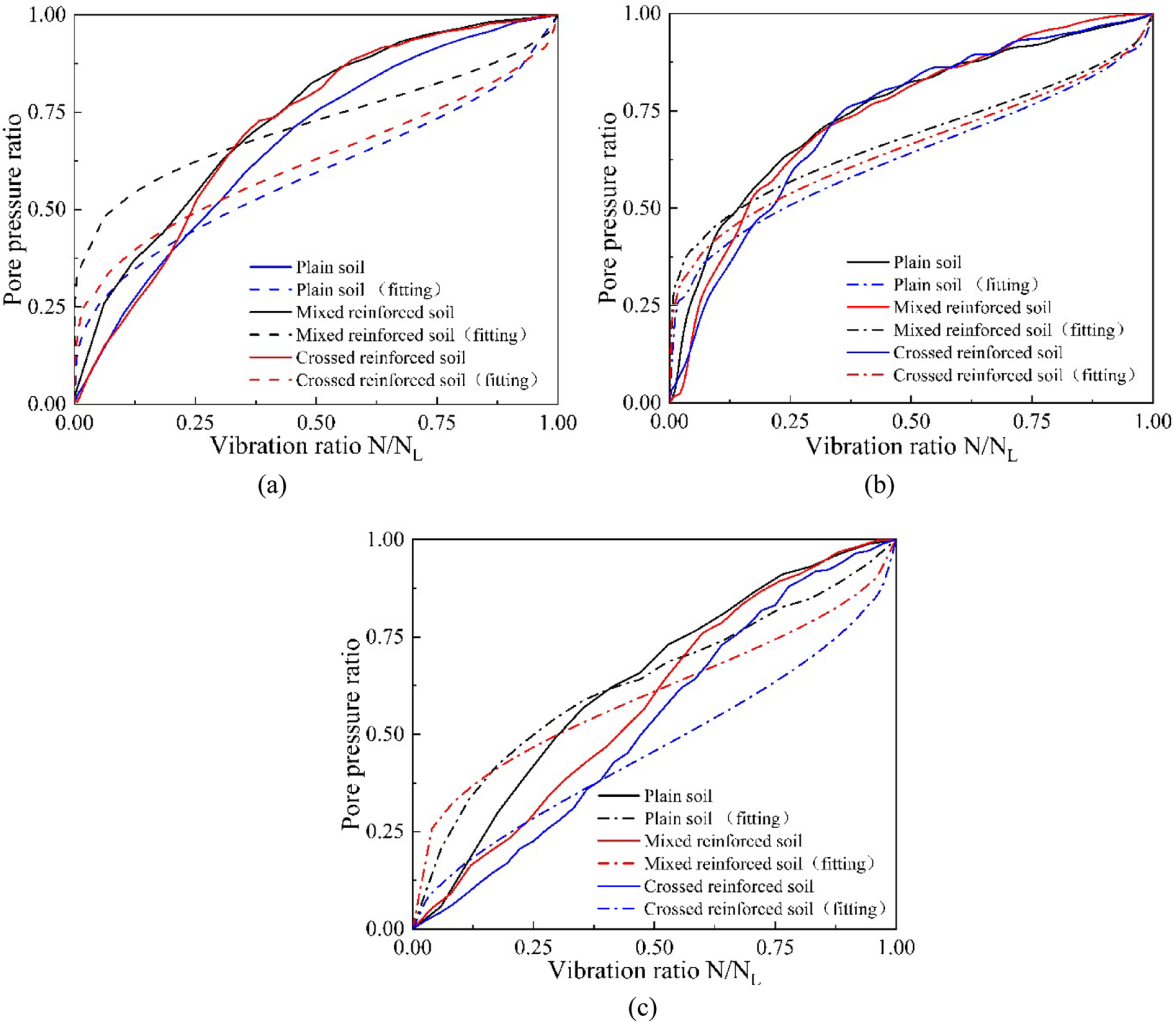
Relationship between pore pressure ratio and vibration ratio computed by Seed model: (**a**) dynamic stress amplitude of 15 kPa, (**b**) dynamic stress amplitude of 20 kPa, and (**c**) dynamic stress amplitude of 25 kPa.

Based on the test data obtained from the dynamic triaxial tests for plain and root-reinforced soils with formula 2, the goodness-of-fit $R^{2}$ and the test constant $a$ of the model were calculated as shown in Table 3. It can be seen that the $R^{2}$ of the seed model in this test is approximately 0.52, which stands for the relatively low fit degree. The average value of parameter *a* is 1.98, with a varied range of 0.83–3.71, which is far from the recommended value of 0.7 in the Seed model. The fitting curves are shown in Fig. 9. To narrow the range of parameter *a* for different specimens, the formula is modified as shown in (3), that is, $b = 1/2a$. Thus, the average value of parameter *b* is 0.46, and the range of parameter *b* variation is 0.13–1.1, narrowing the range of parameter variation and increasing the regularity of the parameter.

3 $$ \frac{u}{{\sigma_{0} }} = \frac{2}{\pi }{\text{arcsin}}\left( {\frac{N}{{N_{L} }}} \right)^{b} $$

**Table 3:** Regression equation calculation results.

Test specimen		Formula						
		$\frac{u}{{\sigma_{0} }} = \frac{2}{\pi }{\text{arcsin}}\left( {\frac{N}{{N_{L} }}} \right)^{\frac{1}{2a}}$		$\frac{u}{{\sigma_{0} }} = \frac{2}{\pi }{\text{arcsin}}\left( {\frac{N}{{N_{L} }}} \right)^{b}$		$\frac{u}{{\sigma_{0} }} = {{\frac{N}{{N_{L} }}} \mathord{\left/ {\vphantom {{\frac{N}{{N_{L} }}} {\left( {c\frac{N}{{N_{L} }} + d} \right)}}} \right. \kern-\nulldelimiterspace} {\left( {c\frac{N}{{N_{L} }} + d} \right)}}$		
–	Dynamic stress	$R^{2}$	$a$	$R^{2}$	$b$	*R*^2^	$c$	$d$
Plain soil	15 kPa	0.6191	2.073	0.6191	0.314	0.9991	0.7256	0.2744
	20 kPa	0.256	3.712	0.256	0.1347	0.9961	0.8603	0.1397
	25 kPa	0.5562	1.558	0.5562	0.3209	0.9956	0.5037	0.4963
Mixed arrangement	15 kPa	0.55	1.924	0.55	0.2414	0.9972	0.5965	0.4035
	20 kPa	0.3505	2.768	0.8898	0.7228	0.997	0.7851	0.2149
	25 kPa	0.6433	1.086	0.6433	0.4604	0.9921	0.2304	0.7696
Crossed arrangement	15 kPa	0.5211	1.598	0.5211	0.2596	0.9924	0.5568	0.4432
	20 kPa	0.4611	2.381	0.9687	1.1571	0.9985	0.6757	0.3243
	25 kPa	0.7337	0.8261	0.7337	0.6053	0.9913	− 0.0079	1.0079
Mean value	–	0.5212	1.982	0.6375	0.4684	0.9955	0.5473	0.4527

**Figure 10:**
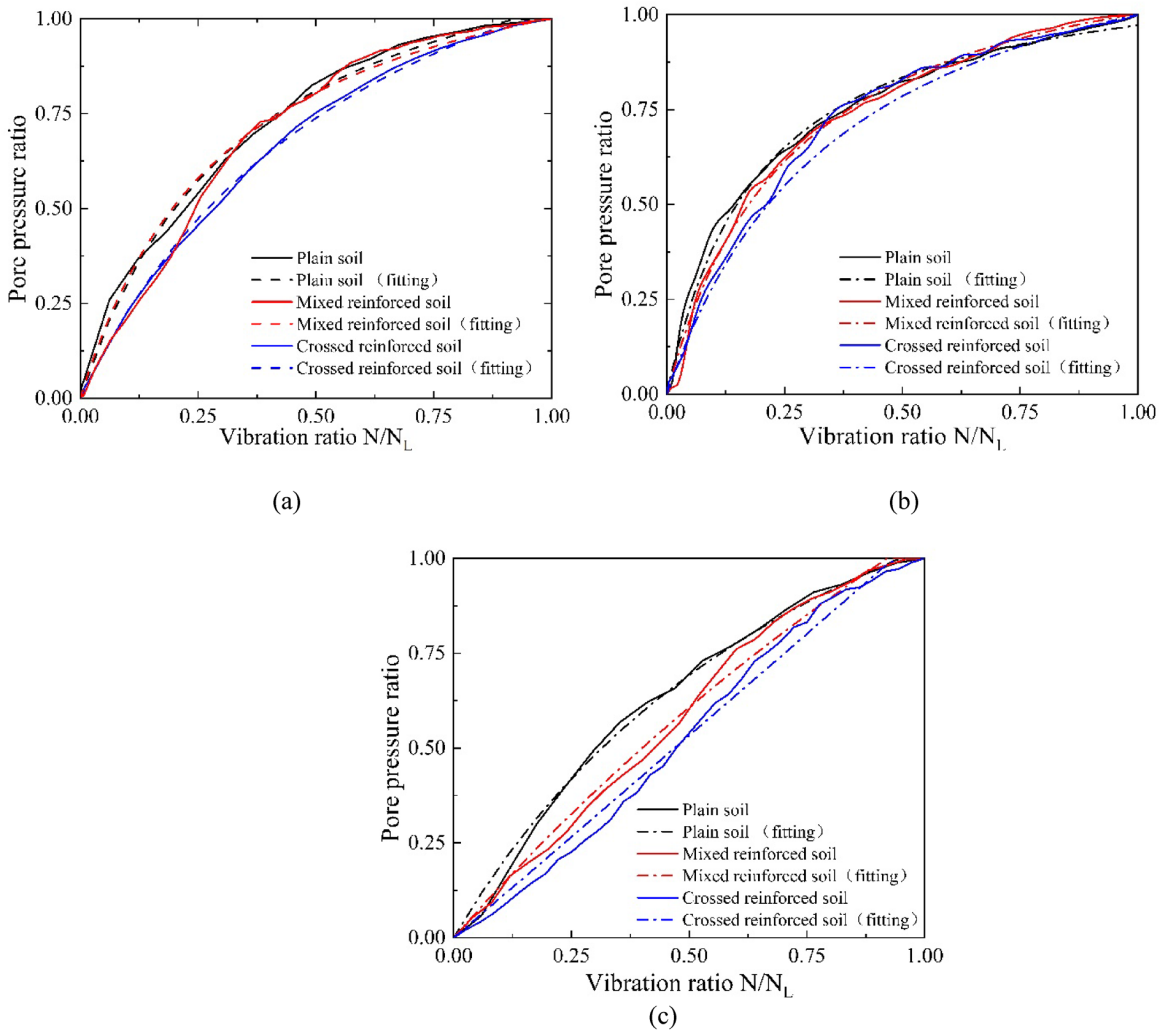
Relationship between pore pressure ratio and vibration ratio computed by proposed model: (**a**) dynamic stress amplitude of 15 kPa, (**b**) dynamic stress amplitude of 20 kPa, and (**c**) dynamic stress amplitude of 25 kPa.

Because the goodness of fit of the seed model is not acceptable, according to the test results, a new pore water pressure development model is proposed, as shown in (4), where *c* and *d* are constant parameters and $$c + d = 1$$ because when the vibration ratio is equal to 1, the pore water pressure ratio should also be equal to 1. As shown in Table 3, the model is more suitable for root-reinforced soil with relatively high $$R^{2}$$. The average values of parameter *c* and parameter $$d $$ are 0.55 and 0.45, respectively, and the range of variation is not large. The fitting curve is shown in Fig. 10.

4 $$ \frac{u}{{\sigma_{0} }} = {{\frac{N}{{N_{L} }}} \mathord{\left/ {\vphantom {{\frac{N}{{N_{L} }}} {\left( {c\frac{N}{{N_{L} }} + d} \right)}}} \right. \kern-\nulldelimiterspace} {\left( {c\frac{N}{{N_{L} }} + d} \right)}} $$

As shown in Table 3, the model proposed above has a better fitting effect than the Seed model. The value of the test constants in the three formulas will influence the shape and the growth rate of the curve. In the Seed model and the new proposed model (4), larger test constants $a$ and $c$ will induce a faster curve growth rate. A larger $b$ in formula (3) will be accompanied by a lower curve growth rate. Under the same load, the root arrangement has a significant effect on the growth rate of the curve. The values of $a$ and $c$ in formula (2) and formula (4) for different soil samples are plain soil > mixed arrangement > crossed arrangement. The growth rate of the plain soil curve is the largest, while that of the crossed reinforced is the smallest. For the same specimen, the critical constant that will affect the curve shape and the growth rate have diverse change rules under loads with high and low dynamic amplitudes. According to Table 3, from low to high loads, the parameters $a$ in formula (2) and *c* in formula (4) both experience an increasing and then decreasing process, which indicates an increase and then a decrease in the curve growth rate. As shown in Fig. 10a,b, with the increase in the dynamic load amplitude from 15 to 20 kPa, the curve slope grows obviously, which demonstrates the acceleration of the pore pressure increase. When applying a load with a higher dynamic load amplitude of 25 kPa, the change in the curve slope does not increase with the load as before but decreases to an extent as shown in Fig. 10c. In summary, root reinforcement can reduce the rate change of pore pressure growth in the soil during liquefaction and make the pore pressure growth rate more stable. With increasing load, the change in the pore pressure growth rate in the liquefaction process will show a trend of increasing first and then decreasing. The test constants in the model depend on the soil type and test conditions. The test constant c in the new model has a smaller range of variation compared to the sed model test constant a, indicating that the new model is more stable in this test. Moreover, the new model (4) has a better fit than the seed model, so the new model is more suitable for root-reinforced soils.

## Discussion

From previous studies, it is well accepted that the root distribution has a strong influence on the strength of the root reinforcement^23^, which has been verified by the test results and analysis in this paper. Regarding the effect of different root distributions on the strength of the soil, the main difference lies in the root direction. Lian, B's study shows that the individual horizontal and vertical arrangement is not as effective as the crossed arrangement because it is reinforced in only one direction compared to the crossed arrangement. Moreover, the reinforcement effect of vertical roots is better than that of horizontal arrangement^24^. In this paper, the diameters and lengths of the root systems added in the experiments were selected according to uniform criteria, and the basic difference between the two root distribution patterns was the proportion of roots in different directions. The proportion of both vertically and horizontally distributed root systems was close to 50% in the cross arrangement. The vertical root system plays a dominant role in resisting deformation. Therefore, the angle of inclination and the relative number of root systems are key factors to be considered when analyzing the reinforcement effect of root distribution. In contrast, the root distribution in the mixed arrangement was random, and the proportion of vertically distributed roots was relatively smaller. Meanwhile, when cyclic loading is applied during the dynamic triaxial test, there is a pressure release process for the specimens, and the root system may rebound during the process. It can be inferred that the crossed arrangement is better than the mixed arrangement for soil reinforcement under cyclic loading because of the different proportions of roots in the vertical direction. However, the development of root stress in root-reinforced soils is complex; only two root distribution patterns were considered in this study, and the quantitative measurements of root distribution were different from those in other studies. Determining how best to select and measure the key characteristic parameters of root distribution is a major challenge that must be overcome in further studies.

In addition to the above test results and analysis, there are certain limitations of this test. As far as soil conditions are concerned, soil saturation, soil type, and soil water content have significant effects on the dynamic properties of root-reinforced soils. Sloping soils in northern China are unsaturated, and current studies of unsaturated soils need to consider the effect of gases in the pores, which is quite a complex situation. In the current mechanical study of unsaturated soils, researchers mainly focus on the water–air interface, and the matric suction in the soil needs to be measured^54–56^. Although most of the soils in nature are unsaturated, for the sake of the convenience of engineering research, the perfect soil, i.e. saturated soil, is usually chosen. Considering the worst case in nature, saturated soil has lower strength and is prone to reach liquefaction than unsaturated soil. Therefore, the traditional triaxial strength test and dynamic triaxial test of soil must be conducted in saturated conditions^57,58^. In practice, when slope soil is subjected to extreme conditions such as rainfall, it will reach saturation, and the strength of the soil is the lowest at this time. We chose saturated soil to conduct the test under the most unfavorable condition of the soil. At present, with the limitation of test equipment, it is more common to study saturated soils instead of unsaturated soils for dynamic triaxial tests of soils under conventional dynamic loads^28–30,59^. Regarding the effects of different soil types. Firstly, different soil types exhibit different dynamic properties under the same test conditions, for example, soils with higher cohesion and density such as silty clay and loess are not susceptible to liquefaction, while soils with low cohesion such as sandy soils are susceptible to liquefaction^29,39,60^. Second, whether the reinforcement effect of roots in soils proposed in this paper can be applied to other soil types. Earlier researchers have verified that the root system has the same reinforcement effect in different soil types under static loads^16,21,22^. There are few studies on root reinforcement in soils under dynamic loads, but studies on soil reinforcement with fibers and geomaterials have shown the versatility of suitable reinforcing materials for soil reinforcement^37,57^. Therefore, the conclusions drawn in this paper should be generalized to different soil types. Soil moisture content has a significant effect on the shear strength of root-reinforced soils under static loading. There exists an optimum soil moisture content that gives the strongest shear strength of root-reinforced soils. Once this value is exceeded, the shear strength decreases as the soil water content increases^13^. The soil moisture content under dynamic loading affects the elastic–plastic deformation and damping ratio of the soil under dynamic loading^61,62^, and the effect on this paper is mainly reflected in the liquefaction state. In this paper, the specimens were saturated with the maximum water content in the soil pores in the context of extreme conditions such as rainfall.

Currently, there is a lack of studies related to unsaturated soils, different soil types, and soil moisture content, which are of great research value in the study of the dynamic properties of root-reinforced soils. This study is a preliminary study of the dynamic behavior of root-reinforced soils. The purpose is to study the changes in dynamic properties of root-reinforced soils under dynamic loads. So we first want to reveal the effect of root distribution on the pattern. The results of the study may vary with soil saturation, soil type, and moisture content. In future research, we will consider some tests on unsaturated soils to be closer to the engineering reality. Also, soil type and soil moisture content should be taken into consideration, and we will supplement the experiments in the next study for further discussion. In addition, considering the test control parameters, in this test, only the dynamic characteristics of the soil at a dynamic load loading frequency of 1 Hz were considered, and the dynamic characteristics of the slope soil at different loading frequencies were not explored. Subsequently, we will continue the research on the effect of load frequency on the dynamic characteristics of the soil. Finally, due to the limitation of specimen preparation, the root arrangement in the root-adding soil still cannot fully simulate the actual root growth distribution, which is also a difficult point to overcome in the field of studying root-adding soil. This is a difficult point to overcome in the field of root-adding soil research. Further research will be conducted to simulate the root distribution in a more realistic way.

## Conclusions

In this study, the effects of different roots in different rows on the deformation characteristics of root–soil complexes were investigated by testing different roots in different rows with different loading forms, and the results obtained were discussed. The following conclusions can be drawn.

1. Under liquefaction conditions, the root system can enhance the dynamic strength of the soil, the enhancement is obvious at low dynamic stress amplitudes, and the crossed arrangement is more effective than the mixed arrangement in enhancing the dynamic strength.
2. By applying cyclic loading, plant roots can improve the damping ratio of the soil and enhance the ability of the soil to resist cyclic loading, and the cross arrangement has a greater damping ratio than the mixed arrangement.
3. A new pore pressure model is proposed, which can more accurately fit the development pattern of pore pressure.
4. The root system significantly enhances the soil liquefaction resistance when suffering a lower dynamic load, and the reinforced effect of the crossed arrangement is better than that of the mixed arrangement. With a larger load, the reinforced effect of the root system weakened.

## Data Availability

The datasets used and/or analysed during the current study are available from the corresponding author on reasonable request.

## References

[1] Zhao H, Tian WP, Li JC, Ma BC (2018). Hazard zoning of trunk highway slope disasters, a case study in Northern Shaanxi, China. Bull. Eng. Geol. Environ..

[2] Hack R, Alkema D, Kruse GAM, Leenders N, Luzi L (2007). Influence of earthquakes on the stability of slopes. Eng. Geol..

[3] Roering JJ, Schmidt KM, Stock JD, Dietrich WE, Montgomery DR (2003). Shallow landsliding, root reinforcement, and the spatial distribution of trees in the Oregon Coast Range. Can. Geotech. J..

[4] Osman N, Barakbah SS (2006). Parameters to predict slope stability—Soil water and root profiles. Ecol. Eng..

[5] Pollen N (2007). Temporal and spatial variability in root reinforcement of streambanks, accounting for soil shear strength and moisture. Catena (Giessen).

[6] Schwarz M, Lehmann P, Or D (2010). Quantifying lateral root reinforcement in steep slopes: From a bundle of roots to tree stands. Earth Surf. Proc. Landf..

[7] Schwarz M, Preti F, Giadrossich F, Lehmann P, Or D (2010). Quantifying the role of vegetation in slope stability: A case study in Tuscany (Italy). Ecol. Eng..

[8] Zhang C (2019). Evaluating the potential slope plants using new method for soil reinforcement program. Catena (Giessen).

[9] Schwarz M, Cohen D, Or D (2011). Pullout tests of root analogs and natural root bundles in soil: Experiments and modeling. J. Geophys. Res. Earth Surf..

[10] Loades KW, Bengough AG, Bransby MF, Hallett PD (2010). Planting density influence on fibrous root reinforcement of soils. Ecol. Eng..

[11] Giadrossich F (2017). Methods to measure the mechanical behaviour of tree roots: A review. Ecol. Eng..

[12] Hossein H, Alireza K (2018). Enhancement of river bank shear strength parameters using Vetiver grass root system. Arab. J. Geosci..

[13] Fan C, Lu JZ, Chen HH (2021). The pullout resistance of plant roots in the field at different soil water conditions and root geometries. CATENA.

[14] Cammeraat E, Beek RV, Kooijman A (2005). Vegetation succession and its consequences for slope stability in SE Spain. Plant Soil..

[15] Abernethy B, Rutherfurd ID (2001). The distribution and strength of riparian tree roots in relation to riverbank reinforcement. Hydrol. Process..

[16] Fan C, Tsai M (2016). Spatial distribution of plant root forces in root-permeated soils subject to shear. Soil Till. Res..

[17] Hairiah K, Widianto W, Suprayogo D, Noordwijk VM (2020). Tree roots anchoring and binding soil: Reducing landslide risk in Indonesian agroforestry. Land (Basel).

[18] Ji X, Cong X, Dai X, Zhang A, Chen L (2018). Studying the mechanical properties of the soil–root interface using the pullout test method. J. Mt. Sci. Engl..

[19] Su L, Hu B, Xie Q, Yu F, Zhang C (2020). Experimental and theoretical study of mechanical properties of root-soil interface for slope protection. J. Mt. Sci. Engl..

[20] Ghestem M (2014). Influence of plant root system morphology and architectural traits on soil shear resistance. Plant Soil..

[21] Meng S, Zhao G, Yang Y (2020). Impact of plant root morphology on rooted-soil shear resistance using triaxial testing. Adv. Civ. Eng..

[22] Lian B, Peng J, Zhan H, Wang X (2019). Mechanical response of root-reinforced loess with various water contents. Soil Till. Res..

[23] Zhang C, Chen L, Liu Y, Ji X, Liu X (2010). Triaxial compression test of soil–root composites to evaluate influence of roots on soil shear strength. Ecol. Eng..

[24] Wang F, Zhao M, Sun Q, Zhang J (2021). Experimental study on shear resistance of Carex-root-fibered soil. Adv. Civ. Eng..

[25] Wright SG, Rathje EM (2003). Triggering mechanisms of slope instability and their relationship to earthquakes and tsunamis. Pure Appl. Geophys..

[26] Cai Y, Chen Y, Cao Z, Sun H, Guo L (2015). Dynamic responses of a saturated poroelastic half-space generated by a moving truck on the uneven pavement. Soil Dyn. Earthq. Eng..

[27] Shirokov VS (2018). Soil and traffic loads on underground pipelines. Soil Mech. Found. Eng..

[28] Tang Y, Zhou J, Liu S, Yang P, Wang J (2011). Test on cyclic creep behavior of mucky clay in Shanghai under step cyclic loading. Environ. Earth Sci..

[29] Li J, Tang Y, Yang P, Liu Q (2015). Dynamic properties of freezing–thawing muddy clay surrounding subway tunnel in Shanghai. Environ. Earth Sci..

[30] Luo J, Miao L (2016). Research on dynamic creep strain and settlement prediction under the subway vibration loading. SpringerPlus.

[31] Lu Z, Fang R, Yao H, Hu Z, Liu AJ (2018). Evaluation and analysis of the traffic load-induced settlement of roads on soft subsoils with low embankments. Sci. Lett. Int. J. Geomech..

[32] Zheng L, Ran F, Yongxiang Z, Hailin Y, Fan G (2019). Study on the dynamic deformation of road high liquid limit subgrade soil. Adv. Civ. Eng..

[33] Rubén G, Hernán P, Svetlana M (2019). Hysteretic behaviour model of soils under cyclic loads. Acta Geophys..

[34] Sitharam TG, GovindaRaju L, Sridharan A (2004). Dynamic properties and liquefaction potential of soils. Curr. Sci. India.

[35] Bray JD, Sancio RB (2006). Assessment of the liquefaction susceptibility of fine-grained soils. J. Geotech. Geoenviron..

[36] Cardile G, Pisano M, Moraci N (2019). The influence of a cyclic loading history on soil-geogrid interaction under pullout condition. Geotext. Geomembr..

[37] Ferreira FB, Vieira CS, Lopes ML, Ferreira PG (2020). HDPE geogrid-residual soil interaction under monotonic and cyclic pullout loading. Geosynth. Int..

[38] Qian JG, Wang QW, Jiang JH, Cai YQ, Huang MS (2018). Centrifuge modeling of a saturated clay ground under cyclic loading. Int. J. Geomech..

[39] Yetis BS (2019). Investigation of the liquefaction potential of fiber-reinforced sand. Geomech. Eng..

[40] Böhm W (1979). Root Parameters and Their Measurement.

[41] Li Y, Wang Y, Wang Y, Ma C (2017). Effects of root spatial distribution on the elastic-plastic properties of soil–root blocks. Sci. Rep..

[42] Yamanouchi, T. & Yauhara, K. Settlement of clay subgrades of low bank roads after opening to traffic. In *Proceedings:Australia-New Zealand Conference on Geomechanics* 115–119 (1975).

[43] Lin JH (2014). Variations in dynamic vehicle load on road pavement. Int. J. Pavement Eng..

[44] Mei G, Qin Q, Lin DJ (2004). Bimodal renewal processes models of highway vehicle loads. Reliab. Eng. Syst. Saf..

[45] Zhao, S. K. Study on deformation mechanism of microscopic structure of soft clay under subway loading. *Master, Tongji University, Shanghai* (2005)

[46] Cai Y, Chen Y, Cao Z, Ren C (2018). A combined method to predict the long-term settlements of roads on soft soil under cyclic traffic loadings. Acta Geotech..

[47] Stewart HE (1986). Permanent strains from cyclic variable-amplitude loadings. J. Geotech. Eng..

[48] Cai Y, Cao XW (1996). Study of the critical dynamic stress and permanent strain of the subgrade-soil under the repeated load. J. Southwest Jiaotong Univ..

[49] Kumar SS, Krishna AM, Dey A (2017). Evaluation of dynamic properties of sandy soil at high cyclic strains. Soil Dyn. Earthq. Eng..

[50] Song DS, Feng Z, Jin HS (2021). Comparison of methods for determining sand dynamic shear modulus and damping ratio. J. Jilin Univ. (Earth Sci. Ed.).

[51] Ren XW, Santamarina JC (2018). The hydraulic conductivity of sediments. A pore size perspective. Eng. Geol..

[52] Ren X (2016). A relation of hydraulic conductivity: Void ratio for soils based on Kozeny–Carman equation. Eng. Geol..

[53] Seed HB, Idriss IM (1971). Simplified procedure for evaluating soil liquefaction potential. J. Soil Mech. Found. Div..

[54] Liu C, Muraleetharan KK (2012). Coupled hydro-mechanical elastoplastic constitutive model for unsaturated sands and silts. I. Formulation. Int. J. Geomech..

[55] Zhao CG, Liu Y, Gao FP (2010). Work and energy equations and the principle of generalized effective stress for unsaturated soils. Int. J. Numer. Anal. Met..

[56] Hamid TB, Miller GA (2009). Shear strength of unsaturated soil interfaces. Can. Geotech. J..

[57] Gong L, Nie L, Liu C, Xu Y (2020). Modelling triaxial tests on fibre-reinforced sands with different fibre orientations using the discrete element method. KSCE J. Civ. Eng..

[58] Michalowski RL, Ermák J (2003). Triaxial compression of sand reinforced with fibers. J. Geotech. Geoenviron..

[59] Qian Y, Han J, Pokharel SK, Parsons RL (2013). Performance of triangular aperture geogrid-reinforced base courses over weak subgrade under cyclic loading. J. Mater. Civ. Eng..

[60] Xiao ZH, Liao HJ, Wen Y, Han B (2011). Accumulative deformation behaviour of loess under cyclic loading. Mater. Res. Innov..

[61] Zhao Y (2021). Dynamic behavior of natural sand soils and fiber reinforced soils in heavy-haul railway embankment under multistage cyclic loading. Transp. Geotech..

[62] Zhang S, Tang C, Zhang X, Zhang Z, Jin J (2015). Cumulative plastic strain of frozen aeolian soil under highway dynamic loading. Cold Reg. Sci. Technol..

